# Supplementary material for: Sarcopenia Screening Allows Identifying High-Risk Patients for Allogenic Stem Cell Transplantation
Source: Cancers (Basel). 2021 Apr 8;13(8):1771. doi: 10.3390/cancers13081771 (PMC8068111; doi:10.3390/cancers13081771)
Supplement: Supplementary file 1 [file cancers-13-01771-s001.pdf]

Sarcopenia Screening Allows Identifying High-Risk Patients for Allogenic Stem Cell Transplantation

Johannes Kirsten, Verena Wais, Sebastian V.W. Schulz, Elisa Sala, Gunnar Treff, Donald Bunjes and Jürgen M. Steinacker

Table S1. Main reasons for mortality in the different groups.

	**Group 1**	**Group 2**	**Group 3**	**Group 4**	**Total**
	(normal)	(impaired Aerobic Capacity	(pre-sarcopenic)	(sarcopenic)	
Infectious diseases	3	9	2	12	26
Relapse	1	3	2	4	10
Graft-versusHost Disease	2	2	1	4	9
Organ failure	0	2	0	1	3
Thrombotic microangiopathy	0	0	1	1	2
other	3	2	2	0	7
